# Pyridinium tri­bromido­stannate(II)

**DOI:** 10.1107/S2414314626010187

**Published:** 2026-10-02

**Authors:** Felix Henkel, Hans Reuter

**Affiliations:** a Chemistry, Osnabrück University, Barabarstr. 7, 49069 Osnabrück, Germany; Vienna University of Technology, Austria

**Keywords:** crystal structure, tri­bromido­stannate ion, tetrel bonds, π–π inter­action, N—H⋯Br hydrogen bonds

## Abstract

Single crystals of the title compound, (C_5_H_6_N)[SnBr_3_] or [PyH][SnBr_3_], were grown from aqueous solutions of pyridinium bromide, [PyH]Br, and tin dibromide, SnBr_2_. The compound crystallizes in the monoclinic space group *P*2_1_/*n* with two formula units in the asymmetric unit. The cations have bond lengths and angles similar to those found in other pyridinium salts. In its first coordination sphere, the anions have a trigonal–pyramidal (*tpy*) shape with the Sn^II^ atoms at the apex and the three bromine atoms at the base. The tin–bromine bond lengths are all greater than 2.5 Å, and the bond angles are all less than 92°, which indicates the presence of *tetrel* bonds. These occur along the twofold screw axis and extend the coordination sphere of the tin atoms to highly distorted, 3_3_-aaa octa­hedra. In the crystal, the two pyridinium cations are aggregated *via* weak π–π inter­actions into centrosymmetric dimers, which form angles of 61.4 (4)° between them. Additional N—H⋯Br hydrogen bonds between cations and anions are bifurcated and weak.

## Abstract 2

The crystal structure of the title salt comprises [PyH]^+^ and [Sn^II^Br_3_]^−^ ions. The structural parameters of the two cations and two anions in the asymmetric unit are presented and discussed in relation to the values in other compounds. Inter­molecular inter­actions such as *tetrel* bonds, π–π inter­actions and N—H⋯Br hydrogen bonds are analysed.

## Graphical abstract

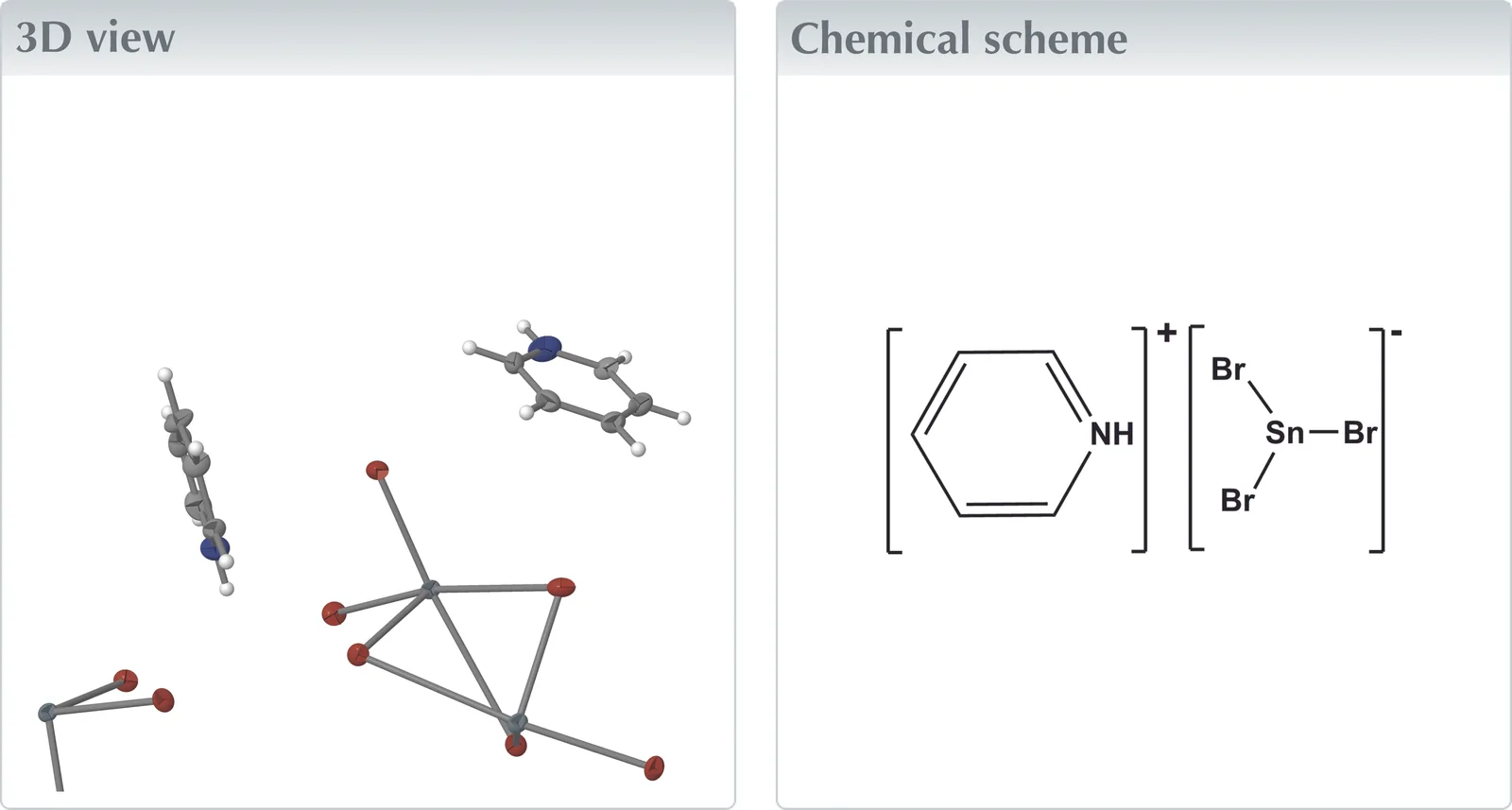

## Structure description

To date, no compounds of divalent tin with pyridinium cations are known. The title compound, pyridinium tri­bromido­stannate(II), is the first example in this class of compounds and, at the same time, provides new insights into the *tetrel*-like inter­action between the anions, on the one hand, and the π–π inter­actions between the cations, on the other.

The title compound crystallizes in the monoclinic space group *P*2_1_/*n* with *Z* = 8 and *Z*′ = 2. Based on the observed inter­nal structural parameters (Fig. 1 ▸), the two [SnBr_3_]^−^ anions do not belong to the class of ‘isolated’ tri­bromido­stannate anions, which are typically characterized by bond angles all larger than 92.7° and atomic distances all shorter than 2.654 Å as they appear in the compounds [Ph_4_P][SnBr_3_] (Müller *et al.*, 1982 ▸), [SnBr(crypt-222][SnBr_3_] (Avery *et al.*, 2012 ▸), or [Cy_3_PH][SnBr_3_] (Naglav *et al.*, 2021 ▸). Rather, they belong to the class of ‘associated’ anions. In this group, one, two or all three tin–bromine bonds are longer than 2.654 Å, and one or more bond angles are less than 90°. Typical examples are: [3-MePipH][SnBr_3_] (Wang *et al.*, 2025 ▸), [C_6_H_10_N_2_][SnBr_3_]_2_ (Matheu *et al.*, 2022 ▸) or [PipH][SnBr_3_] (Abrahams *et al.*, 1994 ▸). In this group of tri­bromido­stannate anions, association occurs *via* the tin atoms, which form additional, so-called *tetrel* bonds (Bauzá *et al.*, 2019 ▸; Brammer *et al.*, 2023 ▸), characterized by the fact that these additional bonds occur in a *trans* position to existing strong, two-centre-two-electron (2*c*–2*e*) bonds. In the broadest sense, *tetrel* bonds can be regarded as extensive three-centre-four-electron (3*c*–4*e*) bonds based on an almost linear alignment of the *p*-orbitals of the three atoms involved in the *X*—Sn⋯*Y* arrangement (Reuter, 2025 ▸).

A look at the crystal structure (Fig. 2 ▸) shows that these inter­molecular inter­actions occur along the *b-*axis direction and are associated with the two twofold screw axes of the space group. This symmetry element, which determines the association of the [SnBr_3_]^−^ ions into chains, is also found, for example, in the compound [3-CF_3_PyH][SnBr_3_] (Chen *et al.*, 2026 ▸). In the case of the anions, the inter­actions result in three *tetrel* bonds (Fig. 3 ▸, Table 1 ▸) for each of the two tin atoms. With the asymmetry quotients *Q* = *d*(Sn⋯Br)/*d*(Sn—Br) listed in Table 1 ▸, these 3*c*–4*e* bonds are highly asymmetric, causing the trigonal–pyramidal, 3_0_ configuration (Schröder *et al.*, 2024 ▸) of the first coordination sphere being extended to a highly distorted octa­hedral, 3_3_-aaa configuration. Similarly, the inter­penetration indices *ρ* (Echeverría & Alvarez, 2023 ▸) using the van der Waals radii [r_vdW_(Br) = 1.83 Å, r_vdW_(Sn) = 2.17 Å; Cordero *et al.*, 2008 ▸] and the covalent radii [r_cov_(Br) = 1.20 Å, r_cov_(Sn) = 1.39 Å); Mantina *et al.*, 2009 ▸] support this concept. Furthermore, the calculation of bond valences (BV) according to Hu *et al.* (2012 ▸) [*R_ij_* = 2.50, *b* = 0.37] shows that the inter­molecular Sn⋯Br distances contribute only slightly [0.26/0.24 valence units for Sn1/Sn2] to the calculated overall bond valence sum [BVS_cal_ = 1.85/1.89] of the divalent tin atoms.

The pyridinium cations are each linked on one side *via* centers of symmetry to form dimers (Fig. 4 ▸). The centers of symmetry are located at Wyckhoff position *c* for the pyridinium ion PyH(1) and Wyckhoff position *d* for the pyridinium ion PyH(2). The least-squares planes of the two pyridinium rings are at an angle of 61.4 (1)° to one another, excluding any additional π–π inter­actions between both. In the dimers, the distance between the least-squares planes is 3.123 Å in the case of PyH(1) and 3.085 Å in the case of PyH(2). Although, or rather because, these distances are relatively short, the overlap occurs essentially only in the region of the hydrogen atoms and not in the region of the ring atoms, so that π–π inter­actions are largely ruled out.

The hydrogen atoms on the nitro­gen atoms of the two pyridinium cations are each involved in hydrogen bonds with two bromine atoms (Table 2 ▸). The donor⋯acceptor distances are quite long and these N—H⋯Br hydrogen bonds are weak, as they are bifurcated (Fig. 5 ▸).

## Synthesis and crystallization

On a Petri dish, a small amount of pyridinium bromide, PyHBr, prepared from pyridine and hydro­bromic acid, was added to a spatula-tip of tin(II) bromide, SnBr_2_, and the mixture covered with two drops of water. This immediately led to the formation of crystals of the title compound in question, which, however, decompose within a very short time.

## Refinement

Crystal data, data collection and structure refinement details are summarized in Table 3 ▸.

## Supplementary Material

Crystal structure: contains datablock(s) I. DOI: 10.1107/S2414314626010187/wm4260sup1.cif

Structure factors: contains datablock(s) I. DOI: 10.1107/S2414314626010187/wm4260Isup2.hkl

CCDC reference: 2592101

Additional supporting information:  crystallographic information; 3D view; checkCIF report

## Figures and Tables

**Figure 1:**
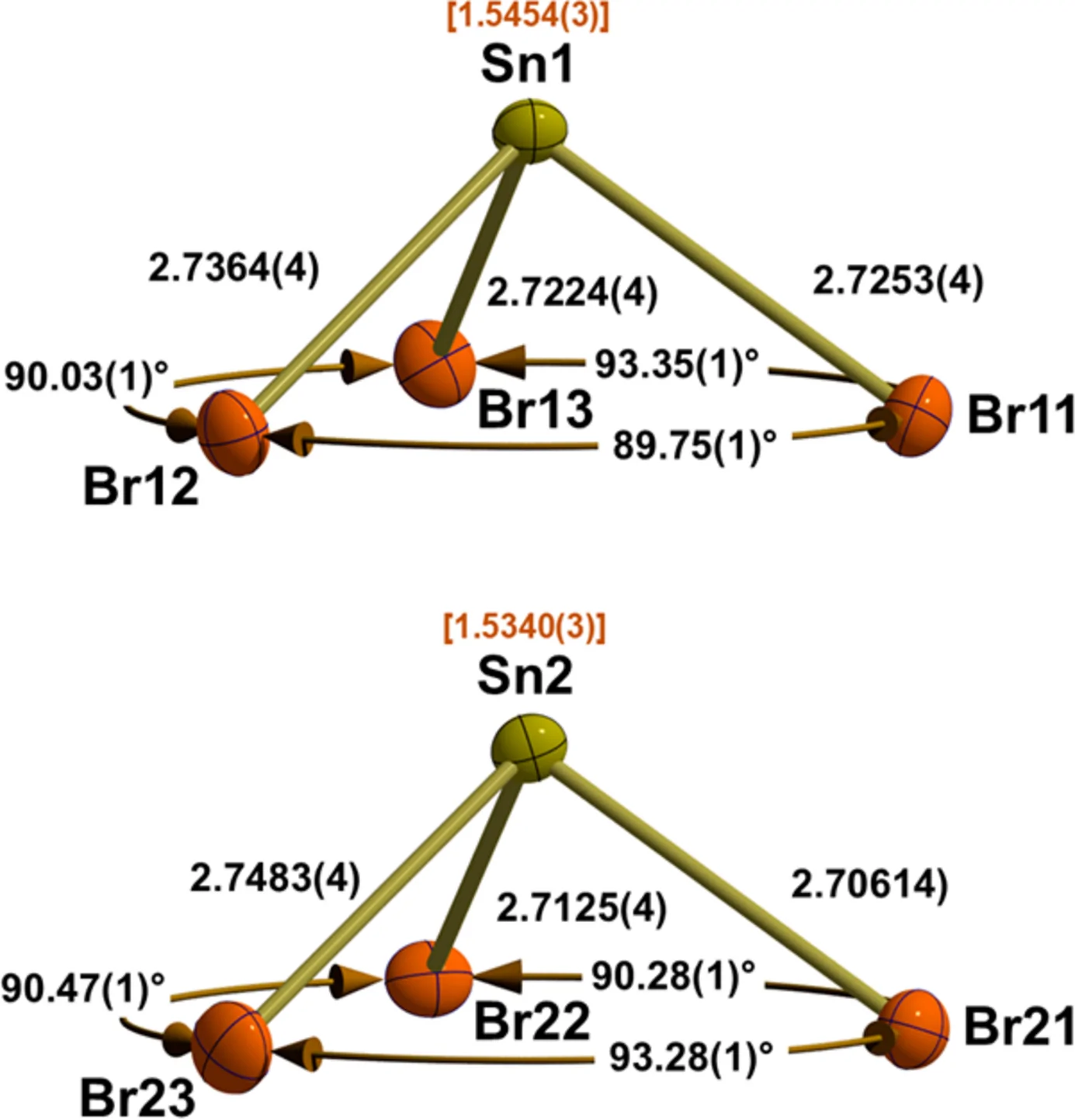
The two [SnBr_3_]^−^ anions of the asymmetric unit with atom numbering, bond lengths (Å), bond angles (°) and distance (Å) of the tin atom from the basal plane in square brackets. All atoms are drawn as anisotropic displacement ellipsoids at the 50% probability level.

**Figure 2:**
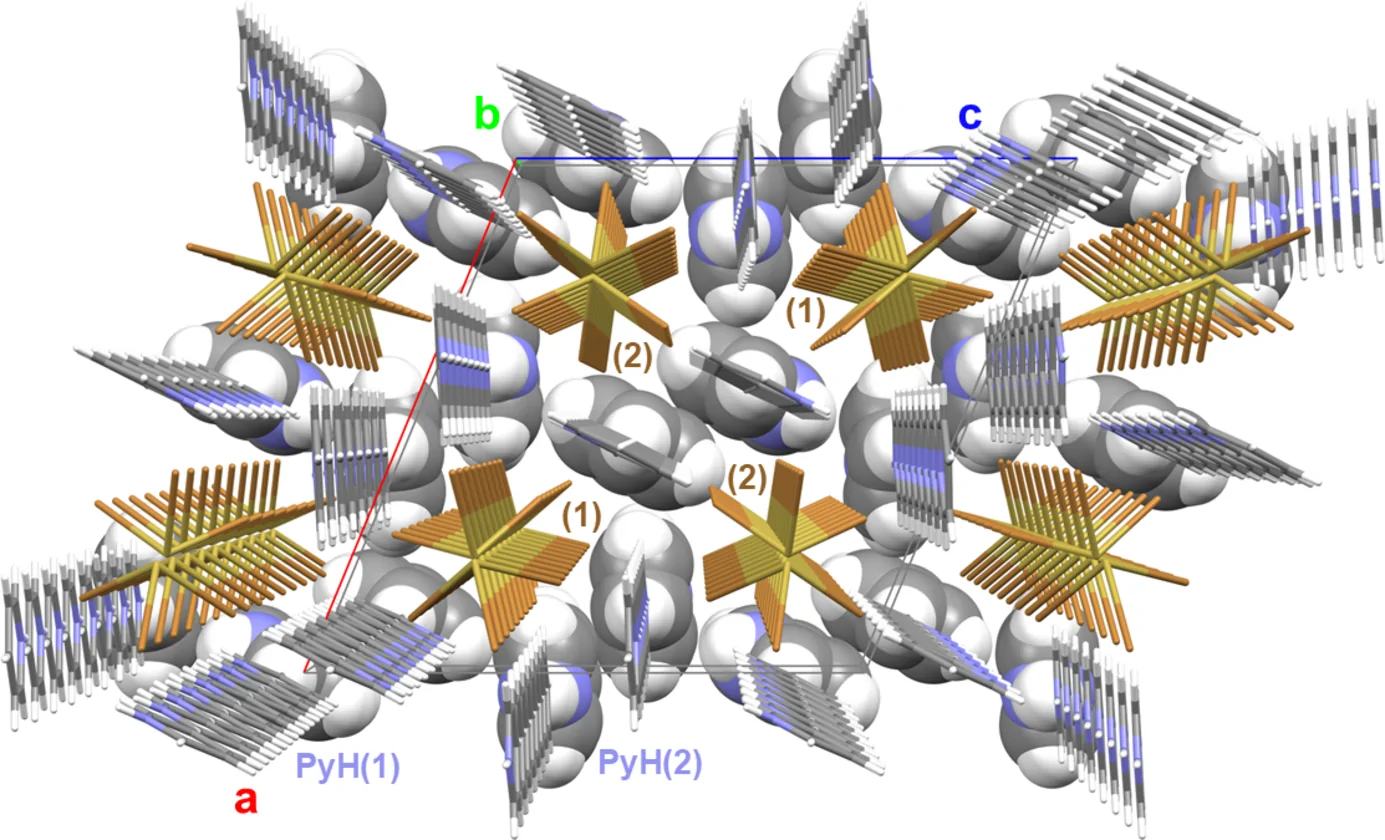
Perspective view into the crystal of [PyH][SnBr_3_] looking down the *b* axis. All components are drawn as stick models with the cations in the background additionally represented as space-filling models. Colour code and van der Waals radii used: Sn = bronze, C = dark grey, 1.70 Å, H = white, 1.20 Å, N = light blue, 1.55 Å, Br = brown.

**Figure 3:**
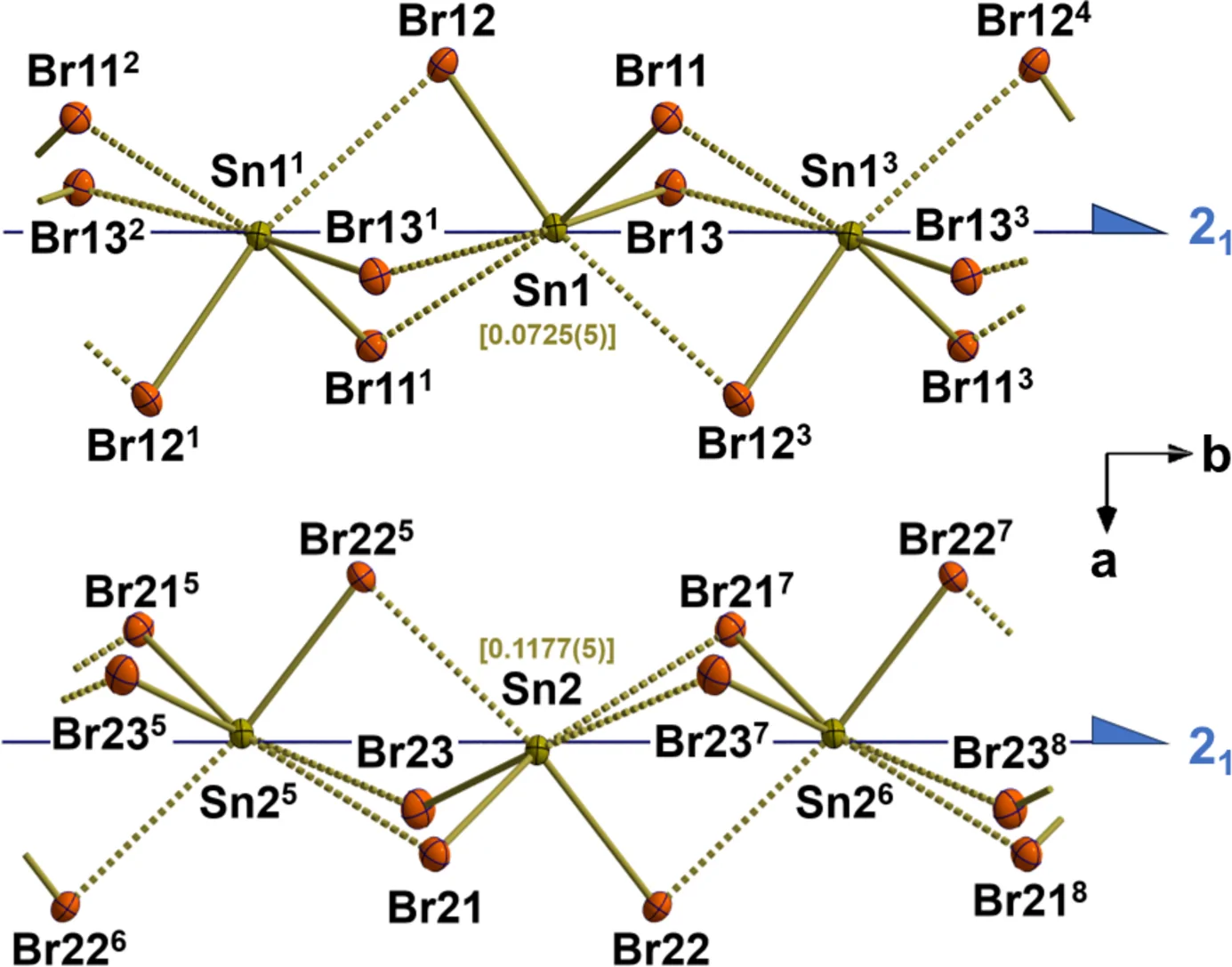
Side-view on the twofold screw axis causing the *tetrel* bond formation in case of Sn1 (top) and Sn2 (bottom**)** with their distances from the screw axis in square brackets**.** Tin-bromine bonds of the first coordination spheres are drawn as solid sticks, those characterizing the tetrel bonds are visualized as dashed sticks. [Symmetry codes: (1) 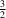 − *x*, −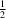 + *y*, 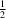 − *z*; (2) *x*, −1 + *y*, *z*; (3) 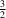 − *x*, 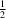 + *y*, 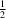 − *z*; (4) *x*, 1 + *y*, *z*; (5) 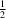 − *x*, −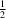 + *y*, 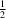 − *z*; (6) *x*, −1 + *y*, *z*; (7) 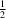 − *x*, 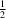 + *y*, 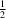 − *z*; (8) *x*, 1 + *y*, *z*.]

**Figure 4:**
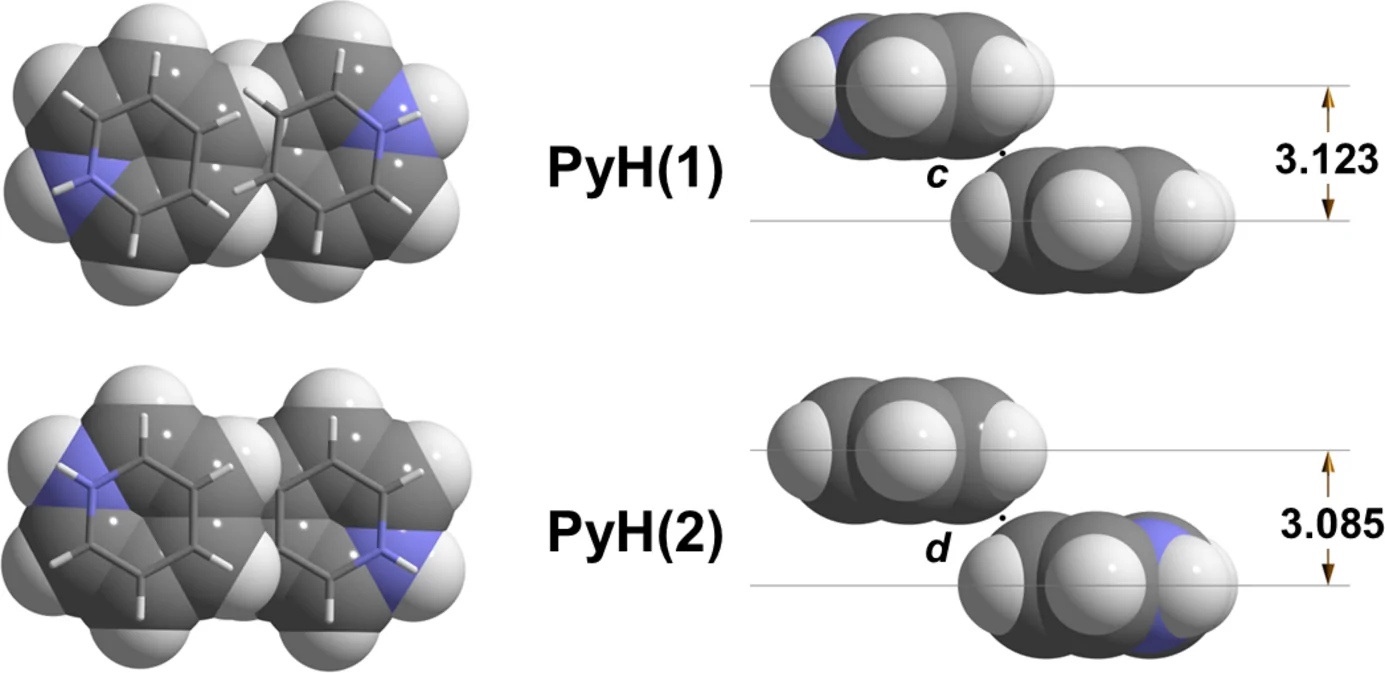
Different representations of the cation stacking. (left) Space-filling model with stick model overlayed viewed perpendicular to the least-squares planes showing the extend of the overlap as resulting from the different centres of symmetry in Wyckhoff positions *c* (PyH1) and *d* (PyH2)*.* (right) Space-filling model of the centrosymmetric dimers in side-view with distances [Å] between the least-squares planes.

**Figure 5:**
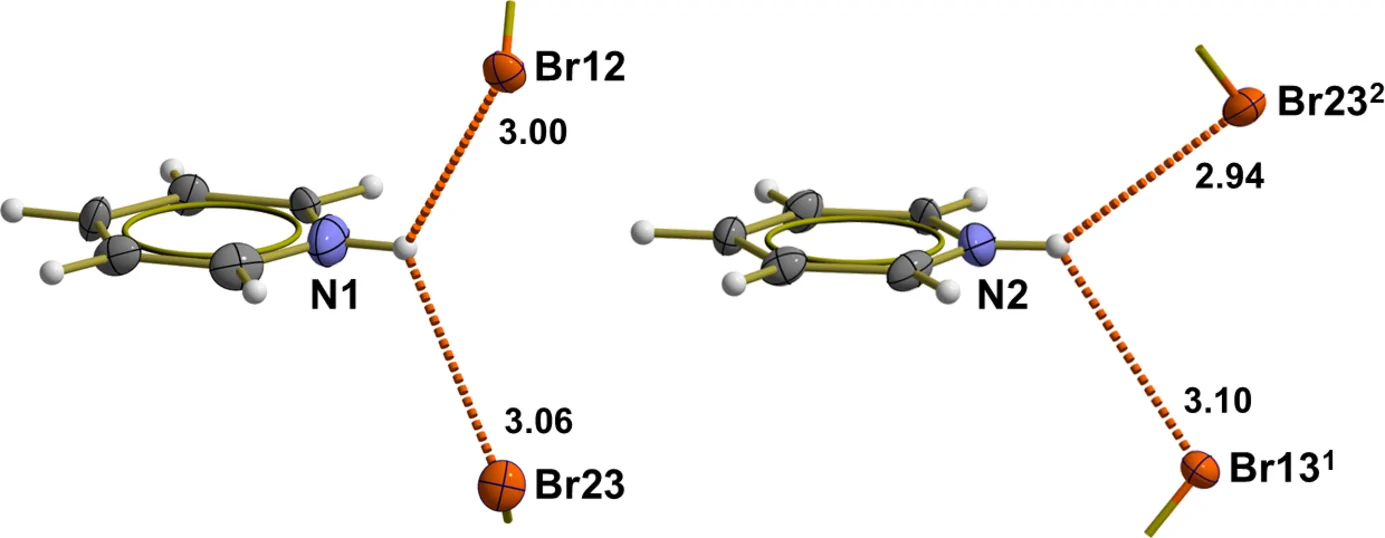
Bifurcated N—H⋯Br hydrogen bonds (dashed sticks, brown) between the pyridinium and tri­bromido­stannate(II) ions with H⋯ Br distances in Å. Br—Sn bonds are indicated as short, two-coloured sticks. All atoms are drawn as anisotropic displacement ellipsoids at the 50% probability level. [Symmetry codes: (1) *x* − 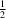, −*y* + 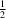, *z* + 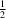; (2) −*x* + 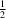, *y* − 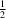, −*z* + 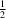.]

**Table 1:** Bond lengths and angles (Å, °), asymmetry parameters *Q*, bond valences BV and inter­penetration indices *p* characterizing the tetrel bonds *X*—-Sn···*Y*.

*X*/*Y*	*d*(Sn—*X*)	*d*(Sn···*Y*)	〈(*X*—Sn···*Y*)	*Q*	BV(Sn···*Y*)	BV(Sn—*X*)	*p*(Sn···*Y*)	〈(Sn—Br···Sn)
Br11/Br11^2^	2.7253 (4)	3.3638 (4)	166.07 (1)	1.23	0.10	0.54	0.45	81.88 (1)
Br12/Br12^1^	2.7364 (4)	3.4665 (4)	167.63 (1)	1.27	0.07	0.53	0.38	79.83 (1)
Br13/Br13^1^	2.7224 (4)	3.3833 (4)	169.03 (1)	1.24	0.09	0.55	0.44	81.56 (1)
sum					0.26	1.62		
Br21/Br21^3^	2.7061 (4)	3.5616 (4)	163.15 (1)	1.32		0.06	0.57	78.57 (1)
Br22/Br22^4^	2.7125 (4)	3.3892 (4)	172.04 (1)	1.25	0.09	0.56	0.43	81.69 (1)
BR31/Br31^3^	2.7483 (4)	3.3749 (4)	170.04 (1)	1.23	0.09	0.51	0.44	81.46 (1)
Sum					0.24	1.65		

Symmetry codes: (1) −*x* + 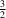, *y* − 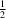, −*z* + 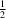; (2) −*x* + 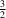, *y* + 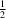, −*z* + 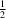; (3) −*x* + 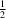, *y* + 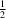, −*z* + 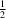; (4) −*x* + 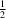, *y* − 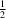, −*z* + 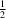.

**Table 2:** Hydrogen-bond geometry (Å, °)

*D*—H⋯*A*	*D*—H	H⋯*A*	*D*⋯*A*	*D*—H⋯*A*
N1—H1⋯Br12	0.88	3.00	3.517 (4)	119
N1—H1⋯Br23	0.88	3.06	3.595 (4)	122
N2—H2⋯Br13^i^	0.88	3.10	3.647 (3)	123
N2—H2⋯Br23^ii^	0.88	2.94	3.623 (3)	136

Symmetry codes: (i) 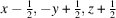; (ii) 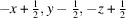.

**Table 3:** Experimental details

Crystal data	
Chemical formula	(C_5_H_6_N)[SnBr_3_]
*M*_r_	438.53
Crystal system, space group	Monoclinic, *P*2_1_/*n*
Temperature (K)	100
*a*, *b*, *c* (Å)	16.3640 (5), 8.0330 (2), 16.4846 (5)
β (°)	112.458 (2)
*V* (Å^3^)	2002.59 (10)
*Z*	8
Radiation type	Mo *K*α
μ (mm^−1^)	14.46
Crystal size (mm)	0.25 × 0.08 × 0.05

Data collection	
Diffractometer	Bruker APEXII CCD
Absorption correction	Multi-scan (*SADABS*; Krause *et al.*, 2015 ▸)
*T*_min_, *T*_max_	0.414, 0.732
No. of measured, independent and observed [*I* > 2σ(*I*)] reflections	171951, 4844, 4040
*R*_int_	0.073
(sin θ/λ)_max_ (Å^−1^)	0.661

Refinement	
*R*[*F*^2^ > 2σ(*F*^2^)], *wR*(*F*^2^), *S*	0.019, 0.040, 1.06
No. of reflections	4844
No. of parameters	183
H-atom treatment	Only H-atom displacement parameters refined
Δρ_max_, Δρ_min_ (e Å^−3^)	0.62, −0.70

Computer programs: *APEX2* and *SAINT* (Bruker, 2009 ▸), *SHELXS* (Sheldrick, 2008 ▸), *SHELXL* (Sheldrick, 2015 ▸), *DIAMOND* (Brandenburg, 2006 ▸), *Mercury* (Macrae *et al.*, 2020 ▸) and *publCIF* (Westrip, 2010 ▸).

## full crystallographic data

### Pyridinium tribromidostannate(II) Crystal data

**Table:**

(C_5_H_6_N)[SnBr_3_]	*F*(000) = 1584
*M**_r_* = 438.53	*D*_x_ = 2.909 Mg m^−^^3^
Monoclinic, *P*2_1_/*n*	Mo *K*α radiation, λ = 0.71073 Å
*a* = 16.3640 (5) Å	Cell parameters from 9978 reflections
*b* = 8.0330 (2) Å	θ = 2.7–27.9°
*c* = 16.4846 (5) Å	µ = 14.46 mm^−^^1^
β = 112.458 (2)°	*T* = 100 K
*V* = 2002.59 (10) Å^3^	Prism, colourless
*Z* = 8	0.25 × 0.08 × 0.05 mm

### Pyridinium tribromidostannate(II) Data collection

**Table:**

Bruker APEXII CCD diffractometer	4040 reflections with *I* > 2σ(*I*)
φ and ω scans	*R*_int_ = 0.073
Absorption correction: multi-scan (*SADABS*; Krause *et al.*, 2015)	θ_max_ = 28.0°, θ_min_ = 1.5°
*T*_min_ = 0.414, *T*_max_ = 0.732	*h* = −21→21
171951 measured reflections	*k* = −10→10
4844 independent reflections	*l* = −21→21

### Pyridinium tribromidostannate(II) Refinement

**Table:**

Refinement on *F*^2^	Primary atom site location: structure-invariant direct methods
Least-squares matrix: full	Hydrogen site location: inferred from neighbouring sites
*R*[*F*^2^ > 2σ(*F*^2^)] = 0.019	Only H-atom displacement parameters refined
*wR*(*F*^2^) = 0.040	*w* = 1/[σ^2^(*F*_o_^2^) + (0.0098*P*)^2^ + 4.8363*P*] where *P* = (*F*_o_^2^ + 2*F*_c_^2^)/3
*S* = 1.06	(Δ/σ)_max_ = 0.001
4844 reflections	Δρ_max_ = 0.62 e Å^−^^3^
183 parameters	Δρ_min_ = −0.70 e Å^−^^3^
0 restraints	

### Pyridinium tribromidostannate(II) Special details

**Table:**

Geometry. All esds (except the esd in the dihedral angle between two l.s. planes) are estimated using the full covariance matrix. The cell esds are taken into account individually in the estimation of esds in distances, angles and torsion angles; correlations between esds in cell parameters are only used when they are defined by crystal symmetry. An approximate (isotropic) treatment of cell esds is used for estimating esds involving l.s. planes.
Refinement. The H atoms were refined in idealized positions and allowed to ride on the parent carbon [*d*(C—H) = 0.95 Å] and nitrogen atoms [*d*(N—H) = 0.88 Å], with one common *U*_iso_(H) factor for each of the two pyridinium cations.

### Pyridinium tribromidostannate(II) Fractional atomic coordinates and isotropic or equivalent isotropic displacement parameters (Å^2^)

**Table:**

	*x*	*y*	*z*	*U*_iso_*/*U*_eq_	
Sn1	0.74639 (2)	0.49613 (3)	0.24574 (2)	0.01520 (5)	
Br11	0.64795 (2)	0.68624 (4)	0.31135 (2)	0.01976 (7)	
Br12	0.60039 (2)	0.30687 (4)	0.15703 (2)	0.02148 (8)	
Br13	0.71023 (2)	0.69213 (4)	0.10145 (2)	0.02061 (7)	
Sn2	0.25755 (2)	0.48288 (3)	0.25112 (2)	0.01560 (5)	
Br21	0.34456 (2)	0.30922 (4)	0.39993 (2)	0.02048 (7)	
Br22	0.39956 (2)	0.68550 (4)	0.28617 (2)	0.01992 (7)	
Br23	0.31740 (2)	0.28145 (4)	0.15074 (2)	0.02476 (8)	
N1	0.5280 (2)	0.2781 (5)	0.3318 (2)	0.0435 (9)	
H1	0.5070	0.3326	0.2817	0.049 (5)*	
C11	0.5631 (2)	0.3630 (4)	0.4065 (2)	0.0235 (8)	
H11	0.5658	0.4811	0.4051	0.049 (5)*	
C12	0.5237 (3)	0.1085 (5)	0.3311 (3)	0.0339 (9)	
H12	0.4985	0.0497	0.2772	0.049 (5)*	
C13	0.5563 (2)	0.0243 (5)	0.4091 (3)	0.0294 (8)	
H13	0.5542	−0.0938	0.4103	0.049 (5)*	
C14	0.5921 (2)	0.1136 (4)	0.4855 (2)	0.0282 (9)	
H14	0.6153	0.0566	0.5401	0.049 (5)*	
C15	0.5948 (2)	0.2826 (4)	0.4841 (2)	0.0257 (8)	
H15	0.6189	0.3436	0.5374	0.049 (5)*	
N2	0.0994 (3)	0.1248 (4)	0.4380 (2)	0.0399 (9)	
H2	0.1048	0.0160	0.4365	0.033 (5)*	
C21	0.1725 (2)	0.2187 (5)	0.4724 (2)	0.0274 (8)	
H21	0.2294	0.1686	0.4958	0.033 (5)*	
C22	0.1632 (2)	0.3898 (5)	0.4730 (2)	0.0271 (8)	
H22	0.2140	0.4586	0.4967	0.033 (5)*	
C23	0.0802 (2)	0.4604 (4)	0.4392 (2)	0.0237 (8)	
H23	0.0733	0.5778	0.4392	0.033 (5)*	
C24	0.0079 (2)	0.3587 (5)	0.4057 (2)	0.0276 (8)	
H24	−0.0497	0.4059	0.3825	0.033 (5)*	
C25	0.0177 (2)	0.1928 (4)	0.4055 (2)	0.0212 (7)	
H25	−0.0328	0.1231	0.3825	0.033 (5)*	

### Pyridinium tribromidostannate(II) Atomic displacement parameters (Å^2^)

**Table:**

	*U*^11^	*U*^22^	*U*^33^	*U*^12^	*U*^13^	*U*^23^
Sn1	0.01591 (10)	0.01134 (10)	0.01766 (11)	0.00070 (8)	0.00562 (8)	0.00067 (9)
Br11	0.02074 (16)	0.02002 (16)	0.02100 (16)	−0.00036 (13)	0.01074 (13)	−0.00295 (13)
Br12	0.02261 (17)	0.01927 (16)	0.02105 (17)	−0.00553 (13)	0.00665 (13)	−0.00294 (13)
Br13	0.02543 (17)	0.02005 (16)	0.01762 (16)	−0.00159 (14)	0.00964 (13)	0.00205 (13)
Sn2	0.01692 (11)	0.01060 (10)	0.01852 (11)	−0.00050 (8)	0.00591 (9)	−0.00026 (9)
Br21	0.02247 (17)	0.01933 (16)	0.02084 (17)	0.00069 (13)	0.00961 (13)	0.00407 (13)
Br22	0.01760 (15)	0.01548 (15)	0.02604 (18)	−0.00319 (12)	0.00764 (13)	0.00006 (13)
Br23	0.03087 (19)	0.02348 (18)	0.01994 (17)	0.00076 (15)	0.00974 (15)	−0.00550 (14)
N1	0.041 (2)	0.059 (3)	0.033 (2)	0.0116 (19)	0.0174 (17)	0.0185 (18)
C11	0.0214 (17)	0.0151 (16)	0.034 (2)	−0.0003 (14)	0.0109 (16)	0.0069 (15)
C12	0.035 (2)	0.043 (2)	0.026 (2)	−0.0012 (19)	0.0137 (18)	−0.0102 (18)
C13	0.0244 (18)	0.0227 (18)	0.039 (2)	−0.0003 (15)	0.0098 (17)	−0.0076 (17)
C14	0.0266 (19)	0.0218 (18)	0.0257 (19)	0.0005 (15)	−0.0020 (16)	0.0065 (15)
C15	0.0270 (19)	0.0205 (17)	0.0245 (19)	−0.0040 (15)	0.0043 (15)	−0.0046 (15)
N2	0.066 (3)	0.0248 (17)	0.0325 (19)	0.0105 (17)	0.0235 (18)	0.0001 (15)
C21	0.0221 (18)	0.034 (2)	0.028 (2)	0.0091 (16)	0.0126 (16)	0.0103 (17)
C22	0.0236 (18)	0.033 (2)	0.027 (2)	−0.0037 (16)	0.0119 (16)	0.0052 (16)
C23	0.0286 (19)	0.0177 (17)	0.0256 (19)	0.0025 (14)	0.0112 (15)	0.0029 (14)
C24	0.0231 (18)	0.0271 (19)	0.028 (2)	0.0020 (15)	0.0053 (16)	0.0055 (16)
C25	0.0198 (16)	0.0171 (16)	0.0184 (17)	−0.0045 (14)	−0.0019 (13)	−0.0025 (14)

### Pyridinium tribromidostannate(II) Geometric parameters (Å, º)

**Table:**

Sn1—Br13	2.7224 (4)	C12—H12	0.9500
Sn1—Br11	2.7253 (4)	C13—C14	1.372 (5)
Sn1—Br12	2.7364 (4)	C13—H13	0.9500
Sn1—Br11^i^	3.3638 (4)	C14—C15	1.359 (5)
Sn1—Br13^i^	3.3833 (4)	C14—H14	0.9500
Sn1—Br12^ii^	3.4665 (4)	C15—H15	0.9500
Sn2—Br21	2.7061 (4)	N2—C21	1.343 (5)
Sn2—Br22	2.7125 (4)	N2—C25	1.350 (5)
Sn2—Br23	2.7483 (4)	N2—H2	0.8800
Sn2—Br23^iii^	3.3749 (4)	C21—C22	1.383 (5)
Sn2—Br22^iv^	3.3892 (4)	C21—H21	0.9500
Sn2—Br21^iii^	3.5616 (4)	C22—C23	1.378 (5)
N1—C11	1.330 (5)	C22—H22	0.9500
N1—C12	1.365 (5)	C23—C24	1.368 (5)
N1—H1	0.8800	C23—H23	0.9500
C11—C15	1.349 (5)	C24—C25	1.343 (5)
C11—H11	0.9500	C24—H24	0.9500
C12—C13	1.368 (5)	C25—H25	0.9500
			
Br13—Sn1—Br11	93.353 (12)	C11—N1—C12	121.5 (4)
Br13—Sn1—Br12	90.025 (12)	C11—N1—H1	119.2
Br11—Sn1—Br12	89.754 (12)	C12—N1—H1	119.2
Br13—Sn1—Br11^i^	98.449 (11)	N1—C11—C15	120.4 (3)
Br12—Sn1—Br11^i^	82.905 (11)	N1—C11—H11	119.8
Br11—Sn1—Br13^i^	95.552 (11)	C15—C11—H11	119.8
Br12—Sn1—Br13^i^	83.623 (11)	N1—C12—C13	119.0 (4)
Br11^i^—Sn1—Br13^i^	71.945 (9)	N1—C12—H12	120.5
Br13—Sn1—Br12^ii^	82.234 (11)	C13—C12—H12	120.5
Br11—Sn1—Br12^ii^	81.127 (11)	C12—C13—C14	118.8 (4)
Br11—Sn1—Br11^i^	166.072 (12)	C12—C13—H13	120.6
Br12—Sn1—Br12^ii^	167.628 (12)	C14—C13—H13	120.6
Br13—Sn1—Br13^i^	169.026 (13)	C15—C14—C13	120.8 (4)
Br11^i^—Sn1—Br12^ii^	107.695 (10)	C15—C14—H14	119.6
Br13^i^—Sn1—Br12^ii^	105.458 (10)	C13—C14—H14	119.6
Sn1—Br11—Sn1^ii^	81.883 (9)	C11—C15—C14	119.5 (3)
Sn1—Br12—Sn1^i^	79.826 (9)	C11—C15—H15	120.3
Sn1—Br13—Sn1^ii^	81.557 (9)	C14—C15—H15	120.3
Br21—Sn2—Br22	90.281 (12)	C21—N2—C25	121.9 (3)
Br21—Sn2—Br23	93.276 (13)	C21—N2—H2	119.1
Br22—Sn2—Br23	90.470 (13)	C25—N2—H2	119.1
Br21—Sn2—Br23^iii^	95.367 (11)	N2—C21—C22	118.5 (3)
Br22—Sn2—Br23^iii^	84.841 (11)	N2—C21—H21	120.7
Br21—Sn2—Br22^iv^	84.321 (11)	C22—C21—H21	120.7
Br21—Sn2—Br21^iii^	163.151 (12)	C23—C22—C21	120.0 (3)
Br22—Sn2—Br22^iv^	172.044 (12)	C23—C22—H22	120.0
Br23—Sn2—Br22^iv^	84.025 (11)	C21—C22—H22	120.0
Br23^iii^—Sn2—Br22^iv^	101.449 (11)	C24—C23—C22	119.0 (3)
Br23—Sn2—Br23^iii^	170.179 (13)	C24—C23—H23	120.5
Br22—Sn2—Br21^iii^	80.923 (10)	C22—C23—H23	120.5
Br23—Sn2—Br21^iii^	101.089 (12)	C25—C24—C23	120.5 (3)
Br23^iii^—Sn2—Br21^iii^	69.679 (9)	C25—C24—H24	119.7
Br22^iv^—Sn2—Br21^iii^	105.736 (9)	C23—C24—H24	119.7
Sn2—Br21—Sn2^iv^	78.569 (8)	C24—C25—N2	120.1 (3)
Sn2—Br22—Sn2^iii^	81.693 (9)	C24—C25—H25	119.9
Sn2—Br23—Sn2^iv^	81.458 (10)	N2—C25—H25	119.9

Symmetry codes: (i) −*x*+3/2, *y*−1/2, −*z*+1/2; (ii) −*x*+3/2, *y*+1/2, −*z*+1/2; (iii) −*x*+1/2, *y*+1/2, −*z*+1/2; (iv) −*x*+1/2, *y*−1/2, −*z*+1/2.

### Pyridinium tribromidostannate(II) Hydrogen-bond geometry (Å, º)

**Table:**

*D*—H···*A*	*D*—H	H···*A*	*D*···*A*	*D*—H···*A*
N1—H1···Br12	0.88	3.00	3.517 (4)	119
N1—H1···Br23	0.88	3.06	3.595 (4)	122
N2—H2···Br13^v^	0.88	3.10	3.647 (3)	123
N2—H2···Br23^iv^	0.88	2.94	3.623 (3)	136

Symmetry codes: (iv) −*x*+1/2, *y*−1/2, −*z*+1/2; (v) *x*−1/2, −*y*+1/2, *z*+1/2.
